# Archaeal MCM Proteins as an Analog for the Eukaryotic Mcm2–7 Helicase to Reveal Essential Features of Structure and Function

**DOI:** 10.1155/2015/305497

**Published:** 2015-10-11

**Authors:** Justin M. Miller, Eric J. Enemark

**Affiliations:** Department of Structural Biology, St. Jude Children's Research Hospital, 262 Danny Thomas Place, Memphis, TN 38105, USA

## Abstract

In eukaryotes, the replicative helicase is the large multisubunit CMG complex consisting of the Mcm2–7 hexameric ring, Cdc45, and the tetrameric GINS complex. The Mcm2–7 ring assembles from six different, related proteins and forms the core of this complex. In archaea, a homologous MCM hexameric ring functions as the replicative helicase at the replication fork. Archaeal MCM proteins form thermostable homohexamers, facilitating their use as models of the eukaryotic Mcm2–7 helicase. Here we review archaeal MCM helicase structure and function and how the archaeal findings relate to the eukaryotic Mcm2–7 ring.

## 1. Introduction

**Figure 1:**
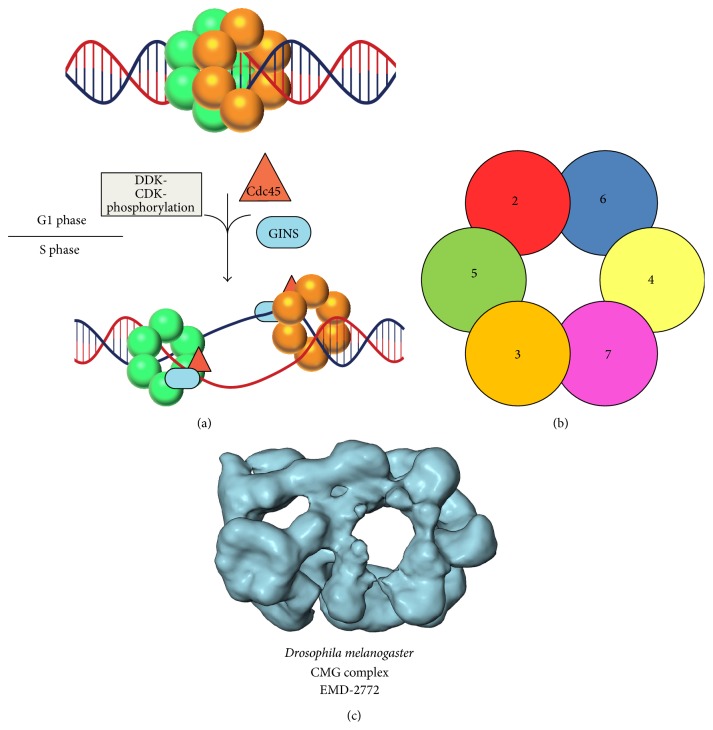
Mcm2–7 activation and organization. (a) Mcm2–7 rings are loaded as inactive double-hexamers at origins of replication by the Origin Recognition Complex (ORC), Cdc6, and Cdt1 (not shown). In a cell-cycle dependent fashion, the Dbf4-dependent Cdc7 kinase (DDK) and cyclin-dependent kinases (CDKs) drive the association of Cdc45 (red triangle) and the GINS complex (blue oval) with the phosphorylated Mcm2–7 ring to yield the active replicative helicase complex, termed the CMG complex (Cdc45-Mcm2–7-GINS). (b) Schematized view of the Mcm2–7 ring from the N-terminal face with subunits labeled to illustrate the order of subunits around the ring [65, 86]. The ring orientation has been selected to correspond with the orientation of the CMG complex shown in (c). (c) The three-dimensional electron microscopy reconstruction of the CMG complex illustrates basic architectural features. The CMG structure representation was prepared with the UCSF Chimera software package [87] and has been labeled with the Electron Microscopy Data Bank (EMDB) accession number.

The process of cell division requires precise duplication of genetic material. This duplication is carried out by replisomes that coordinate multiple protein activities to separate parental DNA strands and to synthesize new strands of complementary DNA. DNA strand separation and also the progression of the replisome along DNA are fueled by a replicative helicase. In eukaryotes, the replicative helicase is a large multiprotein complex, termed the CMG complex, which encircles leading-strand DNA at the replication fork [1–3]. The CMG complex consists of Cdc45, the Mcm2–7 heterohexamer ring, and the GINS tetramer. The Mcm2–7 ring contains six unique gene products, which are loaded with Cdt1 at replication origins by the Origin Recognition Complex (ORC) and Cdc6 [4] to yield two Mcm2–7 rings that inactively encircle the DNA as a double-hexamer [4, 5] (Figure 1(a)). Helicase activation requires the Dbf4-dependent Cdc7 kinase (DDK) and cyclin-dependent kinases (CDKs) to phosphorylate Mcm2–7 and drive recruitment of Cdc45 and the GINS complex. Physical interaction of GINS and Cdc45 with phosphorylated Mcm2–7 supports formation of the active helicase complex [6–13]. Once activated, the two CMG complexes separate and translocate independently in opposite directions on opposing ssDNA strands in the 3′ → 5′ direction to generate two active replication forks [3, 12, 14–18] (Figure 1(a)).

MCM proteins were first identified in a screen for genes essential for minichromosome maintenance in yeast [19]. The identified genes include 6 highly similar proteins with identifiable ATPase motifs [20, 21], Mcm2–7 (Figure 1(b)). The proposed function as the replicative helicase in eukaryotes was initially controversial because Mcm2–7 did not unwind DNA* in vitro*. However, this was resolved by observations that the larger CMG complex unwinds circular and forked double-stranded DNA substrates* in vitro* [2] and that the* Sc*Mcm2–7 complex can unwind DNA by itself when glutamate or acetate was included in the reaction buffer [1].

Electron microscopy studies have revealed general features of Mcm2–7 architecture, including a dynamic gap between the Mcm2 and Mcm5 subunits, an interface previously identified to serve as a “gate” in the ring [1, 22]. EM studies have also shown that Cdc45 and the GINS tetramer associate with the Mcm2–7 hexamer near the Mcm2/5 gate [23, 24], potentially closing the gate (Figure 1(c)). The CMG complex and the Mcm2–7 complex have not been visualized at atomic resolution since each complex has thus far resisted crystallization. A crystal to be used for structure determination must be well ordered, which requires all related molecules to be consistently oriented throughout the crystal. A grossly symmetric ring, such as Mcm2–7, has special crystallization difficulties if the ring is able to adopt multiple orientations that are permuted. These will look similar at the macroscopic level but not at the atomic level. In contrast, archaeal MCM complexes often consist of six identical subunits, and thus all permutations are identical at the macroscopic and atomic level, potentially facilitating crystallization. Indeed, archaeal MCM complexes have been crystallized, which has led to atomic-level descriptions for major functions like DNA binding and ATP hydrolysis. Here we review archaeal MCM helicase structure and function with an emphasis on the advancements in our knowledge of the Mcm2–7 complex that have been derived from archaeal crystallographic studies.

## 2. Archaeal MCM as a Model for Eukaryotic Mcm2–7

As in eukaryotes, the replisomes of archaea centrally consist of a hexameric ring of MCM proteins that exhibits a 3′ → 5′ polarity in* in vitro* double-stranded DNA (dsDNA) unwinding experiments [17, 25, 26]. The amino acid sequences of eukaryotic and archaeal MCM proteins are highly conserved. Due to the strong functional and sequence conservation, archaeal MCM proteins have proven to be powerful tools for elucidating essential features of MCM function. The MCM complexes of many archaea form homohexamers from a single gene product [27]. As such, these archaeal MCM complexes represent simplified versions of the eukaryotic Mcm2–7 complex and can serve as a model, both structurally and biochemically. These models have played a critical role in deciphering essential features of MCM structure and function because exclusively archaeal MCM complexes have generated crystal structures thus far. The identified essential features are likely to be conserved in all MCM complexes, including eukaryotic Mcm2–7.

## 3. MCM Overall Architecture

**Figure 2:**
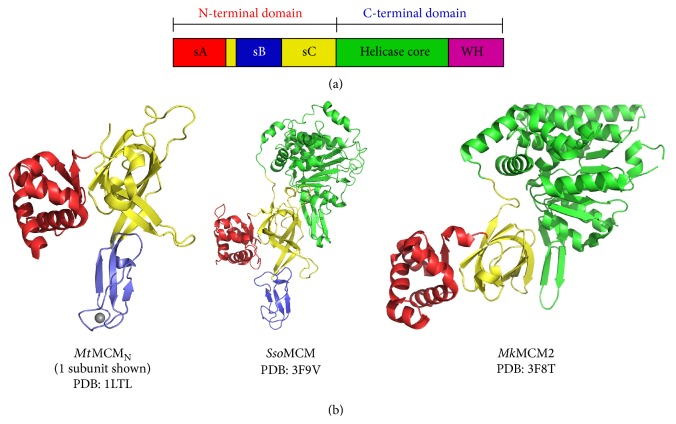
General architecture of MCM proteins. (a) MCM monomers can be subdivided into N- and C-terminal tiers. Each tier can be further subdivided, where the N-terminal half includes subdomains A (red), B (blue), and C (yellow) and the C-terminal domain contains the helicase core (green) and a winged helix domain (magenta). (b) The crystal structures of one subunit of the* Methanobacterium thermoautotrophicum* MCM N-terminal domain double-hexamer (*Mt*MCM_N_, PDB: 1LTL), monomeric* Sulfolobus solfataricus* MCM (*Sso*MCM, PDB: 3F9V), and monomeric* Methanopyrus kandleri* MCM2 (*Mk*MCM2, PDB: 3F8T) each illustrate the general architecture with distinct subdomains A, B, C, and a AAA+ ATPase domain. Subdomains A, B, and C consist of a helical bundle (red), a zinc-binding domain (blue), and an oligonucleotide/oligosaccharide- (OB-) binding fold (yellow), respectively. The AAA+ domain architecture has five *β*-sheets flanked by *α*-helices. All structure representations of Figure 2 were prepared with the Pymol software package [88].

Based on electron microscopy studies, MCM hexamers form a ring with distinct N-terminal and C-terminal tiers [25, 28–31]. Both tiers can independently bind DNA with the N-terminal tier showing a stronger affinity than the C-terminal tier [32]. The C-terminal tier contains the highly conserved AAA+ (ATPases associated with various cellular activities) ATPase/helicase core [33–36] and a winged helix (WH) domain [37] (Figure 2(a)). The C-terminal ATPase tier alone from either* Sso*MCM [32] or* Ape*MCM [38] is sufficient for* in vitro* unwinding of dsDNA. The N-terminal tier (MCM_N_) shows three consistent subdomains in crystal structures, A (sA), B (sB), and C (sC) [39–44] (Figures 2(a)-2(b)), as defined from the crystal structure of *Mt*MCM_N_ [39].

### 3.1. Subdomain A: Peripheral Helical Bundle

Subdomain A is a helical bundle located at the ring periphery and is directly connected to subdomain C by a short linker that may allow for dynamic interaction with the body of the protein [45–48] (Figure 2(b)). Dynamic ring association could play a role in regulating MCM activities, perhaps by restricting access to protein interaction surfaces [44, 46, 49]. Deletion of subdomain A significantly reduces both single-stranded DNA (ssDNA) and dsDNA binding by MCM [28]. The 5′-tail of Y-shaped DNA substrates has been proposed to wrap around the MCM exterior and fit into a binding pocket formed between solvent-exposed C-terminal residues and subdomain A [28, 50–52]. Hence, subdomain A orientation may impact protein:protein interactions, protein:DNA interactions, or both.

The* mcm5-BOB1* P83L mutation in yeast allows cells to bypass the requirement for the S-phase activator protein Cdc7 [53]. The crystal structure of *Mt*MCM_N_ reveals that the corresponding residue sits at the center of the interface between subdomain A and subdomains B/C [39]. A “domain-push” model was proposed where mutation of the buried proline residue to an amino acid with a bulkier side-chain weakens the interaction between subdomain A and subdomains B/C. This hypothesis was tested by introducing P83L, P83W, or P83K Mcm5 mutations in yeast cells [39]. Consistent with the “domain-push” model, bulky side-chains (P83L, P83W, or P83K) allowed S-phase checkpoint bypass, but P83G Mcm5 mutant-containing cells behaved equivalent to wild-type cells. The N-terminal subdomain A has most often been observed in EM and crystal structures to pack against subdomains B/C [39, 40, 43, 45, 48], but subdomain A has also been observed in a different conformation that is extended away from the body of the protein [44, 45, 54]. The “domain-push” may expose a protein interaction surface that is normally only exposed following DDK phosphorylation. Thus, the observation that the* mcm5-BOB1* mutant is able to bypass the S-phase checkpoint may be a result of a shift in protein interaction partners.

### 3.2. Subdomain B: Zinc-Binding Domain

**Figure 3:**
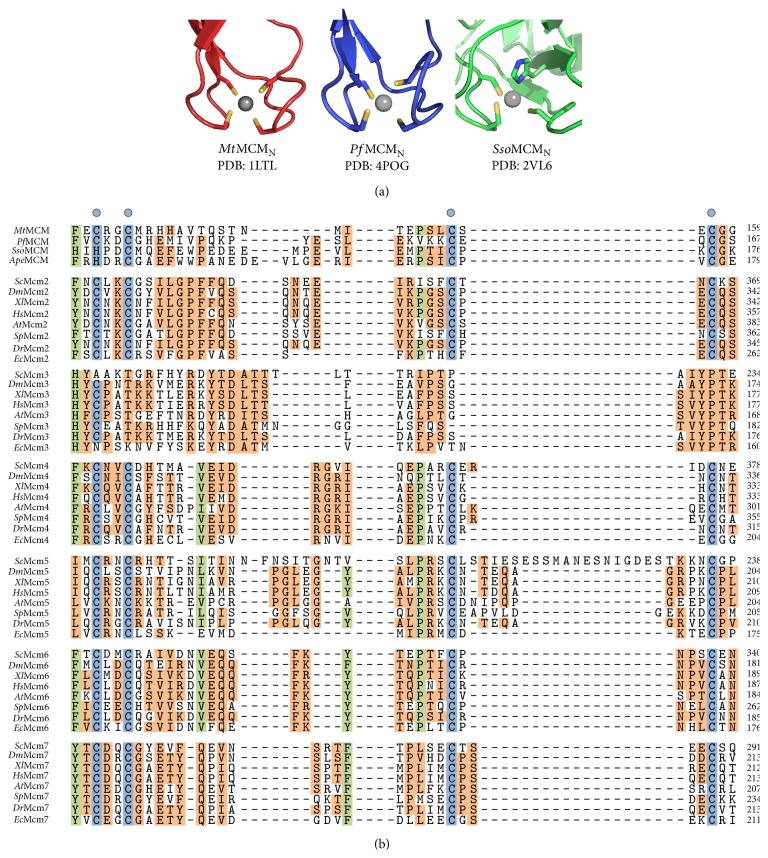
Subdomain B contains a zinc-binding motif. (a) All MCM_N_ X-ray crystal structures contain a zinc atom bound with a tetrahedral geometry. Each structure is depicted in a cartoon representation with zinc-binding side-chains shown in stick. Cysteine sulfurs are colored in yellow, and histidine nitrogen atoms are colored in blue. The remainder of each protein is shown as red, blue, and green for *Mt*MCM_N_ (PDB: 1LTL), *Pf*MCM_N_ (PDB: 4POG), and *Sso*MCM_N_ (PDB: 2VL6), respectively. Zinc atoms are shown as gray spheres. All structure representations of Figure 3 were prepared with the Pymol software package [88]. (b) A multiple sequence alignment shows the strong sequence conservation of the MCM zinc-binding domain. The spacing of the cysteine residues in MCM Zn-binding sequence motifs shows family-specific patterns, where residues displaying a high degree of sequence conservation across all MCM proteins or family-specific sequence conservation have been colored as either green or orange, respectively. Residues involved in zinc-binding are highlighted in blue with a blue dot for emphasis. Mcm3 does not possess an obvious zinc-binding motif, and Mcm5 has an abnormally large spacing between the third and fourth cysteine residues.

All X-ray crystal structures of MCM_N_ reveal a tetrahedrally coordinated zinc ion bound to subdomain B [39, 40, 43] (Figure 3(a)). Critical zinc-binding amino acid side-chains are located on three antiparallel *β*-strands [39–41, 43, 44]. MCM biochemical activities show extreme sensitivity to mutation of the zinc-binding residues, and a cysteine to serine point mutation in* Mt*MCM ablates dsDNA unwinding, DNA-dependent ATPase activity, and ssDNA binding [55]. The zinc-binding sequence motif is commonly CX_2_CX_*n*_CX_2_C (C_4_ type) [39, 43], although a histidine residue is also possible, as observed in the crystal structure of SsoMCM_N_ (Figures 3(a)-3(b)) [40] and in the sequence of* Ape*MCM (Figure 3(b)). Other than Mcm3, all of the subunits of eukaryotic Mcm2–7 possess four highly conserved cysteines that likely coordinate a zinc ion with a tetrahedral geometry. The spacing of the cysteines in these Zn-binding sequence motifs show family-specific patterns (Figure 3(b)). Mcm2 has a CX_2_CX_*n*_CX_2_C motif analogous to those of* Mt*MCM and* Pf*MCM. Moreover, a highly similar CX_2_CX_*n*_CX_2–4_C motif of Mcm4, Mcm6, and Mcm7 also likely binds a zinc ion [55, 56]. In contrast, the four conserved cysteines of Mcm5 have an unusually large spacing between the third and fourth cysteine. The family-specific conservation of the zinc-binding motif, including the lack of an obvious motif in Mcm3, suggests that the zinc-binding sites provide an important mechanism for regulation of eukaryotic Mcm2–7 activities.

### 3.3. Subdomain C: OB-Fold

Subdomain C has an oligonucleotide/oligosaccharide fold (OB-fold), a fold associated with DNA binding [57] (Figure 2(b)). Multiple residues in subdomain C are critical for DNA binding and hexamerization [41, 43, 49, 58]. Further, *β*-loop motifs found in subdomain C appear to facilitate allosteric communication between the N- and C-terminal tiers [59, 60]. A phenylalanine to isoleucine mutation (F345I) in mouse Mcm4 subdomain C has been termed Chaos3 (chromosome aberrations occurring spontaneously 3) [61]. Homozygous Mcm4^Chaos3/Chaos3^ mice develop mammary adenocarcinomas within an average of 12 months for >80% of cases, and mouse cells that possess only F345I mutant Mcm4 (either Mcm4^Chaos3/−^ or Mcm4^Chaos3/Chaos3^) exhibit hypersensitivity to chromosomal breakage [61]. Strains of* Saccharomyces cerevisiae *with the analogous mutation (*Sc*Mcm4 F391I) exhibit the minichromosome loss phenotype typically associated with a loss-of-function for Mcm2–7 [61]. The phenylalanine residue involved is highly conserved as an aromatic residue in all eukaryotic Mcm2–7 subunits and also in* Mt*MCM (*Mt*MCM F170) [61]. The highly conserved aromatic residue is at the center of a hydrophobic intersubunit interface in the structure of* Mt*MCM [39] and is therefore likely critical to the structural integrity of the MCM ring. Although the Chaos3 mutation may not completely prevent interaction of Mcm4 with other Mcm2–7 subunits, the mutation could alter the intersubunit orientations to disrupt DNA binding by the MCM single-stranded binding motif (MSSB, described further below) [43] to generate genomic instability, a general hallmark of cancer cells [62–64].

### 3.4. AAA+ ATPase Core Domain

Crystal structures of MCM proteins containing both N- and C-terminal tiers have revealed a canonical AAA+ ATP binding and hydrolysis site in the C-terminal tier. The MCM AAA+ domain has 5 parallel *β*-strands flanked on either side by *α*-helices [41, 42, 44] (Figure 2(b)). Consistent with other AAA+ family members, such as domain 2 of N-ethylmaleimide sensitive fusion protein, the strand order is *β*5–*β*1–*β*4–*β*3–*β*2, which strictly positions key catalytic residues to generate a competent ATPase site [34, 41, 42, 44]. The MCM AAA+ ATP binding and hydrolysis site contains canonical* cis*- and* trans*-acting elements like the* cis*-acting Walker A and Walker B motifs and the* trans*-acting “SRF” arginine finger motif [65, 66]. Mutation of nearly any residue of the MCM AAA+ active abolishes ATPase activity [32, 66, 67].

### 3.5. Winged Helix-Turn-Helix Domain

Amino acid residues found at the extreme C-terminus of archaeal MCM proteins [37], Mcm6 [68], and the replication factor Mcm10 [69, 70] are predicted to adopt a flexible winged helix-turn-helix fold (Figure 2(a)). For most archaeal MCM proteins, this represents the C-terminal 60–70 amino acids. The NMR structure of the Mcm6 winged helix (WH) domain indicates that the isolated domain is well ordered [68, 71]. However, it is connected to the core of the protein via a flexible linker that allows for mobility of the domain similar to subdomain A [48]. Consistent with this, all X-ray crystal structures containing the C-terminal tier reported to date have been with constructs that lack the WH domain, suggesting that the orientation of this domain is intrinsically flexible [41, 42, 44]. The deletion of the WH region in* Sso*MCM or* Mt*MCM results in a nearly 2-fold enhancement in ATPase activity [32, 72]. For* Sso*MCM, deletion of the WH domain also yields a 15-fold increase in dsDNA unwinding activity [32]. Taken together, the results suggest that the WH domain does not play an essential role during normal unwinding and may instead function during the initial assembly or activation of the helicase or to sense atypical DNA structures, such as damaged DNA, and to signal their presence. Consistent with such a role, this domain is expected to sit directly proximal to the entering dsDNA to be unwound [73].

## 4. MCM Hexamer

**Figure 4:**
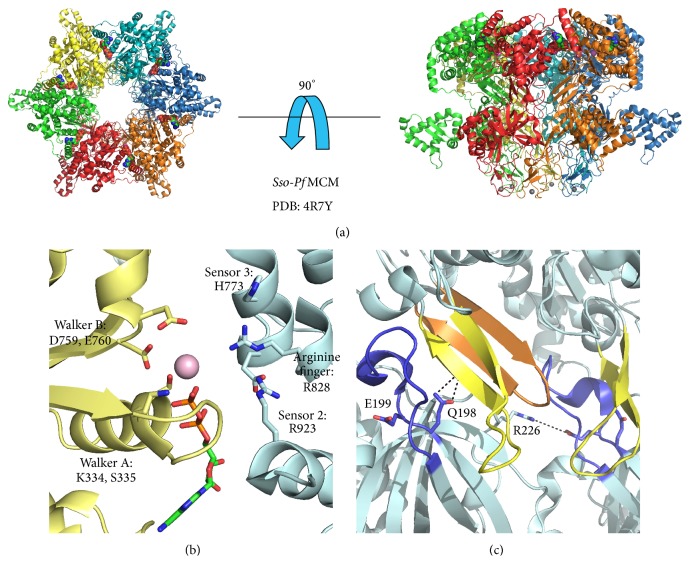
Crystal structure of a nearly full length MCM hexamer. An ADP-bound, nearly full length MCM protein was crystallized by creating a chimeric fusion protein of the N-terminal domain of* Sso*MCM and the C-terminal tier of* Pf*MCM. (a) Views of the* Sso-Pf*MCM hexamer crystal structure parallel and perpendicular to the central channel with each subunit uniquely colored. Magnesium ions are shown as magenta spheres, ADP molecules are shown in space-filling view, and zinc ions are shown as gray spheres. In the view parallel to the central channel, the ATPase domains are projected out of the page. In the perpendicular view, the ATPase domains are located at the top and the N-terminal domains are located on the bottom. (b) The ATPase site is formed at subunit interfaces by* cis*- and* trans*-acting residues shown in stick and labeled. The Walker A and Walker B residues of one subunit are positioned at the left side of the site (yellow), while three basic residues are located on the right side of the site (blue). The bound ADP molecule is shown in stick, and the magnesium ion is represented as a magenta sphere. (c) Residues of the allosteric communication loop (ACL) interact across subunit interfaces and also with main-chain atoms of the helix-2-insert (h2i) *β*-hairpin motif. The ACL is shown in blue and the h2i and presensor-1-*β*-hairpin (ps1*β*) are shown in yellow and orange, respectively. Dashed lines indicate the discussed molecular interactions. All structure representations of Figure 4 were prepared with the Pymol software package [88] and PDB accession code 4R7Y.

Although archaeal MCM proteins are often robust hexamers in solution [32, 66, 73–75], they have proven to be highly resistant to crystallization in a hexameric form. This challenge was overcome by a chimeric fusion of the N-terminal domain of* Sso*MCM with the ATPase domain of* Pf*MCM (*Sso*-*Pf*MCM), which crystallized as a hexamer bound to Mg:ADP [44]. In the* Sso*-*Pf*MCM hexamer structure, the N- and C-terminal domains form distinct tiers stacked on top of one another (Figure 4(a)). The cocrystallized ADP revealed the general architecture of the AAA+ ATP binding site and the specific interactions between the protein and bound nucleotide (Figure 4(b)). A “sensor 3” residue was identified based on the observation of a histidine side-chain that projects into the active site similar to sensor 2 and the arginine finger. The structure also revealed atomic details of the intermolecular interactions between the subunits and also intramolecular interactions between the tiers (Figure 4(c)). In addition, the* Sso*-*Pf*MCM structure crystallized with subdomain A in an extended conformation, in contrast to the previous N-terminal MCM crystal structures with subdomain A packed against subdomains B/C, consistent with an ability for subdomain A to adopt multiple discrete conformations.

## 5. ATPase Site

Consistent with other AAA+ superfamily members, the MCM ATP binding and hydrolysis site is at the interface of two adjacent subunits with catalytic residues contributed both in* cis* and in* trans*. The site is competent to catalyze ATP hydrolysis only when all requisite residues are precisely arranged. In the ADP-bound* Sso*-*Pf*MCM crystal structure, Walker A residue K334 is observed to project into the ATP binding site, and residue S335 coordinates a magnesium cation (Figure 4(b)) [44]. Based on previously proposed AAA+ ATPase catalytic mechanisms, this magnesium cation likely positions the *β*- and *γ*-phosphates of the bound ATP molecule [76] and neutralizes an accumulated negative charge on the *γ*-phosphate during hydrolysis [34, 76]. Based on crystal structures of other AAA+ family members, the second conserved acidic residue of the Walker B motif (E760) is the catalytic base for a water molecule to perform a nucleophilic attack on the *γ*-phosphate of the bound ATP.

The* trans*-acting elements of AAA+ ATPase sites occupy different positions depending on whether ATP, ADP, or no nucleotide is bound to communicate the active site status to the rest of the protein [66, 77, 78]. The* trans*-acting residues must be able to reach the ATP molecule to generate a hydrolysis site with the adjacent subunit. The subunit interface is likely dynamic because the positions of the* trans*-acting residues in the ADP-bound hexameric* Sso*-*Pf*MCM structure are too distant for such interaction, and the subunit interface therefore needs to constrict in order for the* trans*-acting residues to interact with ATP [44, 77].* Trans*-acting MCM residues include “sensor 2” [44, 66, 79], the “arginine finger” [44, 66], and “sensor 3” [44]. The arginine finger may function to polarize the *γ*-phosphate during ATP hydrolysis [76]. The* trans*-acting “sensor 2” element of MCM contrasts most AAA+ ATPases that typically use this motif as a* cis*-acting residue [42, 44, 66, 79].

## 6. MCM Double-Hexamer

**Figure 5:**
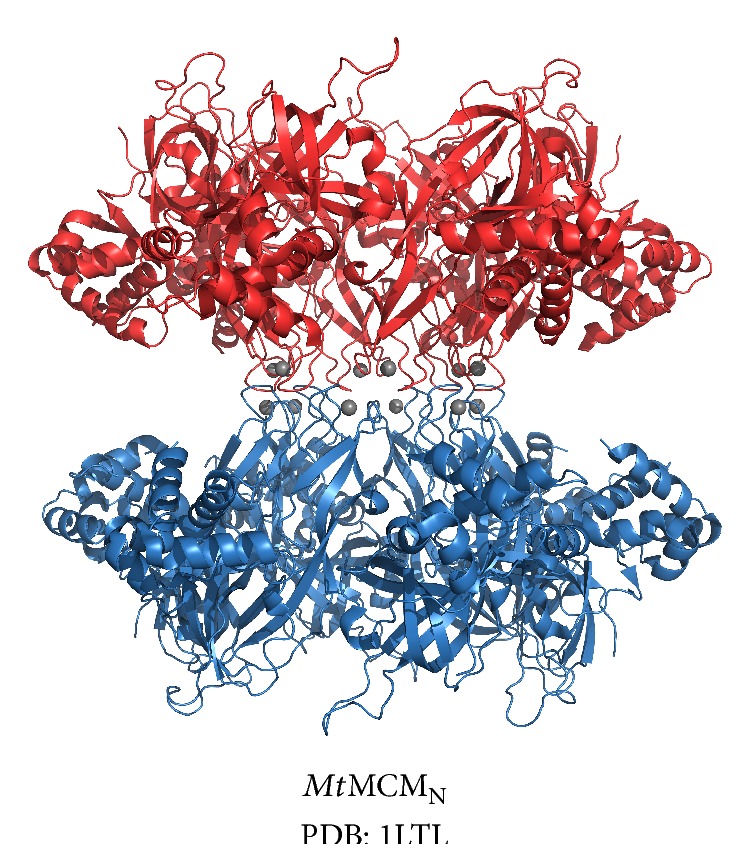
*Mt*MCM_N_ double-hexamer crystal structure. The *Mt*MCM_N_ double-hexamer crystal structure is shown in cartoon representation with one hexamer colored red and the other blue. The two hexamers interact head to head via subdomain B interactions. Zinc atoms are shown as gray spheres. The figure was prepared with the Pymol software package [88] and PDB accession code 1LTL.

In eukaryotes, two Mcm2–7 rings are initially loaded as an inactive double-hexamer at replication origins [4, 5]. The X-ray crystal structure of *Mt*MCM_N_ revealed an MCM double-hexamer with two hexamers arranged head to head via subdomain B domain interactions (Figure 5) [39], providing a model for the Mcm2–7 double-hexamer interaction. A single arginine to alanine mutation at the *Mt*MCM_N_ hexamer:hexamer interface disrupted the interaction in favor of single hexamers [80]. A double-hexamer structure had previously been observed for full length* Mt*MCM in scanning transmission electron microscopy images [25]. In contrast to the stable double-hexamer of* Mt*MCM, X-ray crystal structures of *Sso*MCM_N_ and *Pf*MCM_N_ consist of single hexamers [40, 43]. Although a double-hexamer structure has not yet been universally detected among archaeal MCM complexes, double-hexamer architecture is likely to be conserved when MCM rings are first loaded onto DNA prior to the onset of bidirectional replication [81].

## 7. DNA Binding

The N-terminal and C-terminal domains of MCM are independently able to bind DNA with several modules that line the interior channel of the hexameric ring. These modules and their interaction with DNA are detailed further below.

### 7.1. MCM Single-Stranded Binding Motif (MSSB)

**Figure 6:**
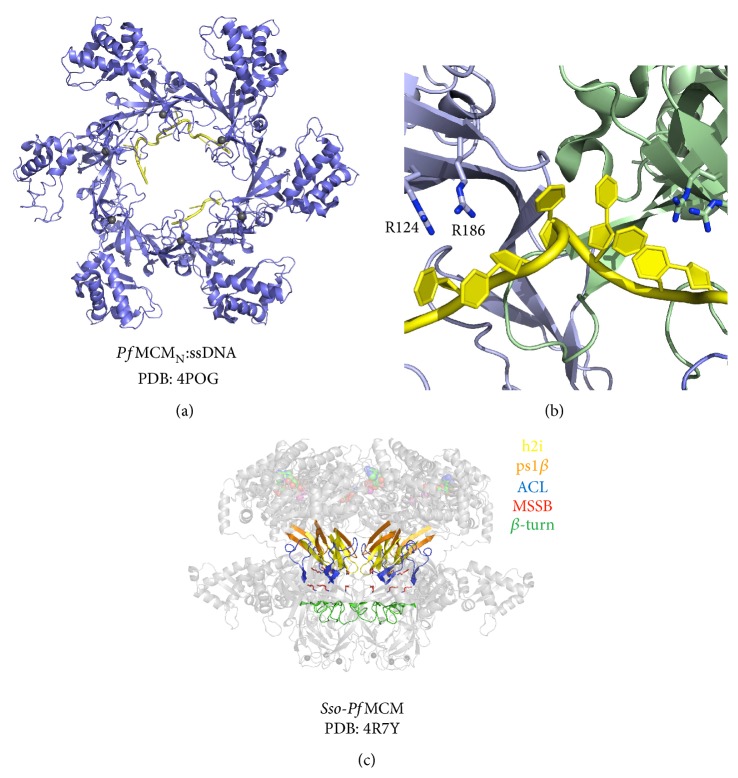
Features of DNA binding in MCM hexamers. (a) The cocrystal structure of *Pf*MCM_N_ bound to ssDNA revealed that DNA binds in the plane of the hexamer. The DNA binds with a 3′ to 5′ polarity in the clockwise direction around the ring when viewed from the N-terminal side of the hexamer. DNA is shown in yellow and zinc ions are shown as grey spheres. (b) DNA binds across subunit interfaces through interactions between the DNA and MCM Single-Strand Binding (MSSB) residues R186 and R124 (*Pf*MCM_N_:ssDNA structure, PDB: 4POG), shown in stick. (c) Important residues of the central channel in the MCM hexamer structure (PDB: 4R7Y) with other components transparent. The h2i, ps1*β*, ACL, MSSB, and N-terminal *β*-turn motifs are shown in yellow, orange, blue, red, and green, respectively. All structure representations of Figure 6 were prepared with the Pymol software package [88].

The MCM N-terminal domain binds ssDNA as revealed by a recent X-ray crystal structure of *Pf*MCM_N_ bound to ssDNA [43]. In this structure, ssDNA binds in the plane of the ring, rather than perpendicular as would be expected if DNA were threaded through the central channel (Figure 6(a)) [43]. This DNA binding configuration contrasts the configurations previously seen for the nucleic acid complexes of the hexameric helicases E1 [77], DnaB [82], and Rho [83] where nucleic acid binds perpendicular to the plane of the hexamer. When viewed from the N-terminal side of the complex (Figure 6(a)), the ssDNA binds with 3′ to 5′ polarity in the clockwise direction around the ring. The MCM_N_:ssDNA structure revealed that 4 ssDNA nucleotides are bound per subunit, which also contrasts with other hexameric helicases that bind either one (E1 [77] and Rho [83]) or two (DnaB [82]) nucleotides per subunit. Interestingly, the MCM_N_:ssDNA structure reveals that ssDNA is bound across subunit interfaces but is not bound at all interfaces of a hexamer (Figure 6(b)) [43]. The intersubunit distance appears to dictate whether ssDNA can bind at a particular subunit interface with ssDNA observed only at tighter interfaces [43].

The *Pf*MCM_N_:ssDNA cocrystal structure revealed specific amino acid residues of the OB-fold subdomain C that bind to ssDNA (Figure 6(b)). This motif is termed the MCM Single-Strand Binding (MSSB) motif (Figure 6(b)) and forms a positively charged DNA binding pocket near the interface of the N- and C-terminal tiers (Figure 6(c)). Residues R124 and R186 were demonstrated to most significantly impair DNA binding where the affinity of R124A and R186A point mutants for ssDNA decreased 7- and 6-fold, respectively [43]. The R124A/R186A double-mutant exhibits a 25-fold reduction in ssDNA binding affinity with much higher protein concentrations required for observation of binding relative to the wild-type protein. Mutation of similarly positioned residues in* Sso*MCM abolishes dsDNA unwinding activity [74].

In* Saccharomyces cerevisiae*, the* Pf*MCM R124 and R186 MSSB residues are conserved as either an arginine or lysine in Mcm4, Mcm6, and Mcm7 [43]. In Mcm2, Mcm3, and Mcm5, only one of the two positively charged residues is conserved but not both. Interestingly, complementation studies in yeast demonstrated that while single-subunit MSSB mutants are viable, cells with two-subunit MSSB mutations are lethal: Mcm4/Mcm6, Mcm4/Mcm7, and Mcm6/Mcm7 [43] (Figure 1(b)). In fact, any of these combinations leave the Mcm2–7 ring defective in helicase loading and severely defective for replication [43]. These data suggest a role for the MSSB during Mcm2–7 helicase loading and activation.

### 7.2. N-Terminal *β*-Turn

A second N-terminal motif implicated in DNA interaction is a *β*-turn that projects into the central channel of the hexamer (Figure 6(c)). In* Sso*MCM, this motif spans residues 241–251 (polypeptide sequence QDSPVKRGSRA) between *β*-strands *β*11 and *β*12 [40]. The *β*-turn is present in all archaeal MCM proteins and has been studied in* Sc*Mcm4 and* Sc*Mcm5 [39, 40, 43, 84]. The length and sequences of this loop vary among Mcm2–7 subunits. In X-ray crystal structures of archaeal MCM hexamers, the N-terminal *β*-turn is the narrowest part of the central channel, varying from 17 Å to 23 Å [39, 40, 43]. Despite poor sequence conservation in this region, the presence of positively charged residues on the *β*-turn is required for DNA binding [39, 73]. Double-mutation of two positively charged residues (K246A/R247A) on the tip of the N-terminal *β*-turn in* Sso*MCM leads to an 8-fold reduction in DNA binding [73]. Similarly, DNA binding is abolished for the comparable alanine double-mutant of* Mt*MCM (R226A/228A) [39]. Based on these experiments, the DNA binding activity attributed to the N-terminal *β*-turn is proposed to play a critical role in DNA loading [73, 84]. Structure-guided yeast genetic approaches have suggested that the *β*-turn of* Sc*Mcm5 is important for binding to origins of replication and subsequent initiation of DNA replication [84]. Thus, the N-terminal *β*-turn likely has a major role in facilitating initial DNA binding.

### 7.3. Presensor-1 *β*-Hairpin (ps1*β*) and Helix-2-Insert (h2i)

**Figure 7:**
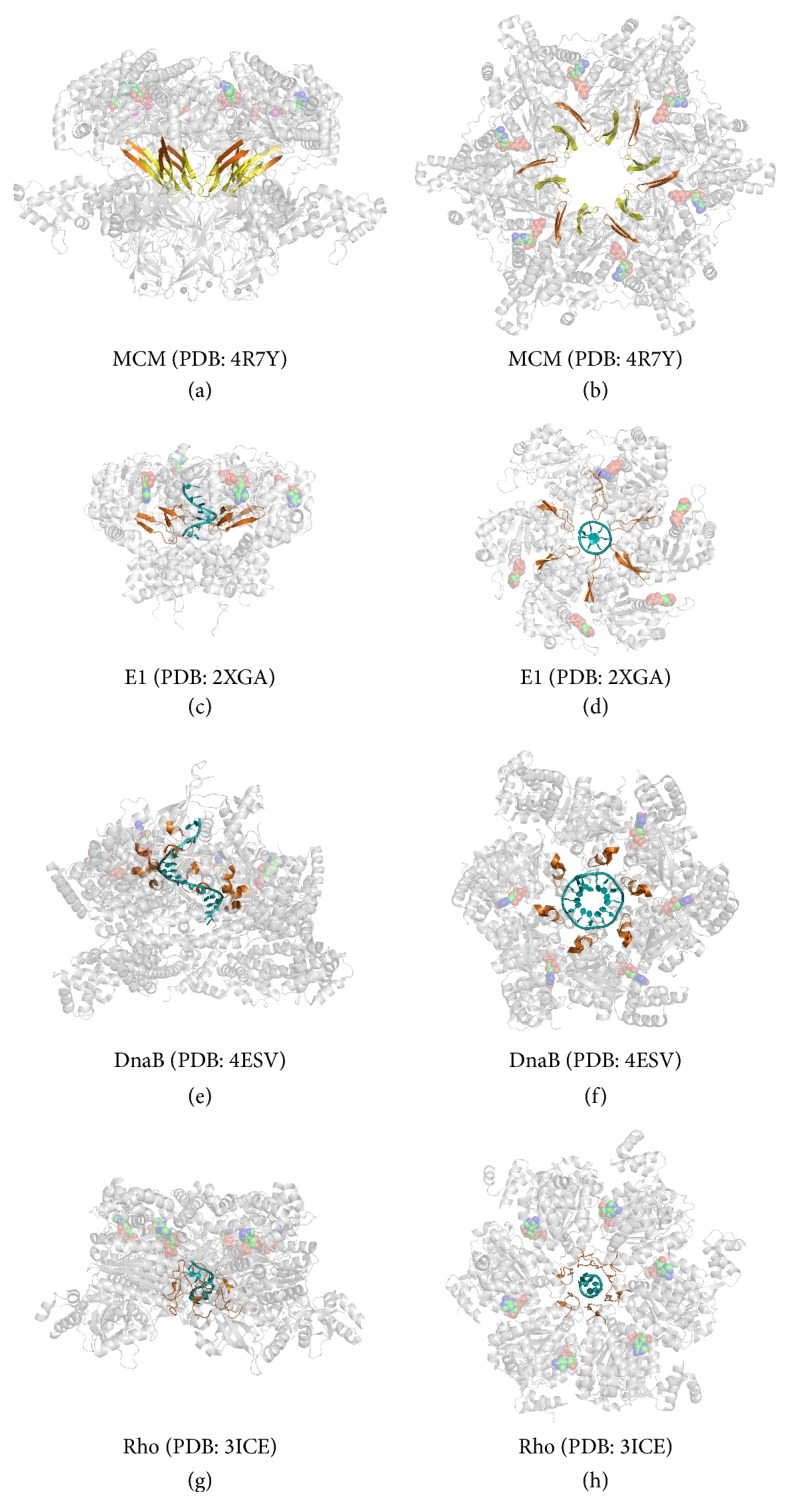
Comparison of MCM with representative members of other hexameric helicase superfamilies. The hexameric MCM structure viewed either perpendicular (a) or parallel (b) to the central channel axis. The MCM h2i and ps1*β* central channel modules are colored yellow and orange, respectively. (c–h) Comparison with representative members of other helicase superfamilies such as E1 ((c-d), SFIII, PDB: 2XGA), DnaB ((e-f), SFIV, PDB: 4ESV), and Rho ((g-h), SFV, PDB: 3ICE) suggests that MCM central channel modules will move “up” and “down” to translocate DNA. In the crystal structures of each of these hexameric helicases, basic residues on loops projected into the central channel interact with nucleic acid in a spiral-staircase-like arrangement. In this mode of binding, the central channel loops from adjacent subunits occupy different heights around the ring to form a “spiral-staircase” shape. The movement of DNA is then achieved by the movement of the central channel loops. DNA is colored in teal and DNA binding loops are colored orange. All structures are viewed either perpendicular (left column) or parallel (right column) to the central channel axis. All structure representations of Figure 7 were prepared with the Pymol software package [88].

The ATPase domain of each MCM monomer contains two *β*-hairpin motifs that project into the central channel of the hexamer, the helix-2-insert (h2i), and the presensor-1-*β*-hairpin (ps1*β*) (Figures 7(a)-7(b)) [41, 42, 44, 72]. The h2i motif is defined by a *β*-*α*-*β* insertion in primary sequence between Walker A and Walker B motifs [35, 72, 79]. This feature is found as a unique insert within helix-2 of the conserved ASCE (additional strand catalytic glutamate) ATPase family [35, 72, 85]. Similarly, the ps1*β* feature represents an insertion between sensor 1 motif and the preceding helix [35]. Within the AAA+ superfamily, the ps1*β* superclade includes the SFIII helicases, HslU, ClpX, Lon, ClpA, MCM, dynein, midasin, YifB, and many others [35]. The ps1*β* superclade is subdivided into HslU/ClpX/Lon/ClpAB-C and h2i subclades, where MCM proteins belong to the h2i subclade since they contain both the ps1*β* and the h2i motifs [35].

MCM mutants lacking either the ps1*β* or h2i motifs are unable to catalyze dsDNA unwinding, despite still being competent for DNA binding [72, 73]. The ps1*β*-deletion mutant displays a weakened binding affinity for Y-shaped DNA of 343 nM versus 152 nM for wild-type protein [73]. Interestingly, this contrasts an h2i-deletion mutant, which displays a shift in binding affinity for a blunt dsDNA substrate from greater than 1 *μ*M for the wild-type protein to ~8 nM for the h2i-deleted mutant [72]. The observation of tighter binding for an h2i-deleted MCM protein may suggest a role for the h2i in destabilizing DNA:protein interactions, which could facilitate the movement of DNA during unwinding. The primary role of the ps1*β* motif is most likely to bind DNA since deletion of the ps1*β* feature yields the weakened affinity expected for a motif intimately involved in DNA binding. The lack of detectable unwinding activity for either the ps1*β*- or h2i-deletion mutants indicates that both C-terminal *β*-hairpins are essential.

## 8. DNA Translocation

The conformation of the h2i and ps1*β* motifs has been proposed to depend on whether ATP or ADP is bound [41, 42, 72]. In the ADP-bound MCM hexamer structure, these loops are observed to be in a “down” position (Figure 7(a)) [44]. Thus, a view perpendicular to the central channel of the hexamer defines the C-terminal domain face as the “up” direction and the N-terminal face as the “down” direction. The orientation of the MCM hexamer shown in Figure 7(a) would result in DNA being translocated with a net movement from the top to the bottom face. Consequently, this motion implies that the h2i and ps1*β* motifs would be in the “up” position in the ATP-bound state. Following hydrolysis of ATP to ADP, the loops would move to the “down” position shown in Figure 7(a). This direction of DNA translocation is consistent with earlier FRET studies showing that MCM hexamers bind forked DNA with the C-terminus facing the leading direction [73].

The projection of each C-terminal *β*-hairpin into the central channel of the MCM hexamer is expected to position positively charged residues for interaction with DNA (Figure 7(b)). This is conceptually similar to other hexameric helicases such as E1 [77], DnaB [82], and Rho [83]. In the crystal structures of each of these hexameric helicases, positive residues on loops projected into the central channel interact with nucleic acid in a spiral-staircase-like arrangement (Figures 7(c)–7(h)). In this mode of binding, the central channel loops from adjacent subunits occupy different heights around the ring to form a “spiral-staircase” shape. The movement of DNA is then achieved by the concerted movement of the entire staircase of loops [77, 82, 83]. As an example, the adjacent subunits of the E1 structure adopt ATP-like, ADP-like, and apo forms around the ring that clearly demonstrate that the loop positions depend on whether ATP, ADP, or no nucleotide is bound at the associated ATP binding site [77]. Basic features of this mode of DNA binding and translocation are most likely shared by all the hexameric helicase superfamilies: SFIII (E1), SFIV (DnaB), SFV (Rho), and SFVI (MCM) [89].

Although MCM proteins display high homology with SFIII helicases, the presence of both the h2i and ps1*β* features in MCM proteins suggests significantly different mechanisms for coupling the energy of ATP binding and hydrolysis to dsDNA unwinding. Deletion of the h2i *β*-hairpin in* Mt*MCM results in ~12-fold increase in dsDNA-stimulated ATPase activity relative to wild-type* Mt*MCM [72]. However, no detectable dsDNA unwinding is observed for the h2i-deletion mutant, despite the observation that the h2i-deleted mutant still binds both ssDNA and dsDNA [72]. Taken together, these observations suggest that the deletion of the h2i motif actually disrupts the coupling of ATP binding and hydrolysis to DNA unwinding such that the helicase can bind dsDNA and also hydrolyze ATP but cannot unwind dsDNA. This is conceptually analogous to an automobile engine shifted to neutral with deletion of the h2i motif disengaging the MCM motor from dsDNA unwinding.

### 8.1. Allosteric Communication Loop

While the C-terminal helicase core can catalyze unwinding of dsDNA in the absence of the MCM N-terminal tier, inclusion of the N-terminal tier results in an increase in the processivity of MCM catalyzed dsDNA unwinding [32]. Such increases in processivity are observed whether the two halves are expressed as a single gene product or when each half is expressed separately and incubated together during unwinding experiments [32]. An important interaction between each half is mediated through a conserved loop found at the interface of the N- and C-terminal tiers termed the “allosteric communication loop” (ACL, Figure 6(c)). Mutational studies have revealed that point mutations in this loop can have either stimulatory or inhibitory effects on the observed MCM dsDNA unwinding activity [60]. For the majority of point mutations introduced to this loop, the rate of* Mt*MCM catalyzed dsDNA unwinding is observed to decrease relative to the wild-type protein, but mutation of either Q181 or E185 to alanine actually results in an increase in unwinding rate. Deletion of the entire ACL causes a loss of unwinding activity. However, activity is restored by the additional deletion of the N-terminal *β*-turn, which suggests the position of the N-terminal *β*-turn could be controlled by the ATP hydrolysis cycle via the ACL [59]. In the* Sso-Pf*MCM hexamer crystal structure, a fully conserved glutamine residue (homologous to* Mt*MCM Q181) at the C-terminal end of the ACL interacts with the h2i *β*-hairpin of the C-terminal AAA+ domain (Figure 4(c)). This glutamine:h2i interaction and the enhanced unwinding rate observed for* Mt*MCM Q181A are mutually consistent with an interaction that restricts interdomain movement and thus regulates helicase activity [60]. When the interaction is removed by mutation of the glutamine to alanine, the helicase activity is no longer regulated and can occur at an increased rate.

Additional roles for the ACL in the regulation of MCM catalyzed helicase activity are found in the observation that* Mt*MCM E182R mutation results in a 7-fold decrease in unwinding rate relative to wild-type* Mt*MCM for unwinding of forked DNA substrates [60]. In the* Sso*-*Pf*MCM structure, the homologous glutamate residue (*Sso-Pf*MCM E199) interacts with an arginine residue of an adjacent subunit,* Sso-Pf*MCM R226 [44] (Figure 4(c)). Thus, the* Mt*MCM E182R mutation would disrupt a salt bridge between adjacent subunits and likely increase the distance between subunits by electrostatic repulsion. Furthermore, the ACL is observed to project towards the AAA+ ps1*β* motif of an adjacent subunit, which could be disrupted in the* Mt*MCM E182R mutant due to an altered intersubunit distance. The proximity of the ACL to the h2i of the same subunit and the ps1*β* of a neighboring subunit has previously been shown by Double Electron-Electron Resonance (DEER) spectroscopy, with the ACL:ps1*β* distance estimated to be approximately 30 Å [59]. While the role of the ACL:ps1*β* interaction is not clear, it may function like the ACL glutamine:h2i interaction to stabilize the ps1*β* and regulate MCM helicase activity. Thus, multiple roles have emerged for the ACL, including regulation of helicase activity, maintenance of intersubunit interfaces, and intrasubunit interactions.

## 9. Concluding Comments

Much like any machine, the MCM helicase requires specific events to occur in a precise order to unwind dsDNA. The identification of the essential events and their timing is complicated in the eukaryotic Mcm2–7 complex because the subunits do not equivalently interact, and they also interact with Cdc45 and the subunits of the GINS complex. For this reason, archaeal MCM proteins have emerged as useful models for elucidating the essential features of the complex interaction network present in MCM hexamers. A thorough understanding of the many interactions within the replisome is essential to understand the replication fork and DNA replication. Atomic resolution crystal structures will continue to reveal mechanistically important conformational states of the MCM complex, including how MCM hexamers specifically respond to binding different nucleotides, oligonucleotides, and protein interaction partners. As new information becomes available, both for the structural/biochemical details of MCM function and for disease-associated mutations, the translation of basic science information will undoubtedly aid in the treatment of primary disease states.
